# Supplementary material for: Metabolite Profiles of the Relationship between Body Mass Index (BMI) Milestones and Metabolic Risk during Early Adolescence
Source: Metabolites. 2020 Jul 31;10(8):316. doi: 10.3390/metabo10080316 (PMC7464362; doi:10.3390/metabo10080316)
Supplement: Supplementary file 1 [file metabolites-10-00316-s001.pdf]

Supplementary Materials

Detailed Metabolon Global Metabolomic Platform Materials and Methods

Sample Preparation for Global Metabolomics. Samples were stored at –80°C until processed. Sample preparation was carried out as described previously [53] at Metabolon, Inc. Briefly, recovery standards were added prior to the first step in the extraction process for quality control purposes. To remove protein, dissociate small molecules bound to protein or trapped in the precipitated protein matrix, and to recover chemically diverse metabolites, proteins were precipitated with methanol under vigorous shaking for 2 min (Glen Mills Genogrinder 2000) followed by centrifugation. The resulting extract was divided into five fractions: two (i.e., early and late eluting compounds) for analysis by ultra-high performance liquid chromatography-tandem mass spectrometry (UPLC-MS/MS; positive ionization), one for analysis by UPLC-MS/MS (negative ionization), one for the UPLC-MS/MS polar platform (negative ionization), and one sample was reserved for backup.

Three types of controls were analyzed in concert with the experimental samples: samples generated from a pool of human plasma extensively characterized by Metabolon, Inc. or generated from a small portion of each experimental sample of interest served as technical replicate throughout the data set; extracted water samples served as process blanks; and a cocktail of standards spiked into every analyzed sample allowed instrument performance monitoring. Instrument variability was determined by calculating the median relative standard deviation (RSD) for the standards that were added to each sample prior to injection into the mass spectrometers (median RSD for the current study was 5%; $n \geq 30$ standards). Overall process variability was determined by calculating the median RSD for all endogenous metabolites (i.e., non-instrument standards) present in 100% of the pooled human plasma or client matrix samples (median RSD for the current study was 10% based on >400 hundred metabolites). Experimental samples and controls were randomized across the platform run.

Mass Spectrometry Analysis. Extracts were subjected to non-targeted UPLC-MS/MS, as described [51]. As part of Metabolon's general practice, all columns were purchased from a single manufacturer's lot at the outset of experiments. All solvents were similarly purchased in bulk from a single manufacturer's lot in sufficient

quantity to complete all related experiments. For each sample, vacuum-dried samples were dissolved in injection solvent containing eight or more injection standards at fixed concentrations, depending on the platform. The internal standards were used both to assure injection and chromatographic consistency. Instruments were tuned and calibrated for mass resolution and mass accuracy daily.

The UPLC-MS/MS platform utilized a Waters Acquity UPLC with Waters UPLC BEH C18-2.1×100 mm, 1.7 µm columns and a Thermo Scientific Q-Exactive high resolution/accurate mass spectrometer interfaced with a heated electrospray ionization (HESI-II) source and Orbitrap mass analyzer operated at 35,000 mass resolution. The sample extract was dried then reconstituted in acidic or basic LC-compatible solvents, each of which contained 8 or more injection standards at fixed concentrations to ensure injection and chromatographic consistency. One aliquot was analyzed using acidic, positive ion-optimized conditions and the other using basic, negative ion-optimized conditions in two independent injections using separate dedicated columns (Waters UPLC BEH C18-2.1x100 mm, 1.7 µm). Extracts reconstituted in acidic conditions were gradient eluted using water and methanol containing 0.1% formic acid, while the basic extracts, which also used water/methanol, contained 6.5mM ammonium bicarbonate. A third aliquot was analyzed via negative ionization following elution from a HILIC column (Waters UPLC BEH Amide 2.1x150 mm, 1.7 µm) using a gradient consisting of water and acetonitrile with 10mM Ammonium Formate. The MS analysis alternated between MS and data-dependent MS^2 scans using dynamic exclusion, and the scan range was from 80-1000 *m/z*.

Compound Identification, Quantification, and Data Curation. Metabolites were identified by automated comparison of the ion features in the experimental samples to a reference library of chemical standard entries that included retention time, molecular weight (m/z), preferred adducts, and in-source fragments as well as associated MS spectra and curated by visual inspection for quality control using software developed at Metabolon [52]. Identification of known chemical entities is based on comparison to metabolomic library entries of purified standards. Commercially available purified standard compounds have been acquired and registered into LIMS for determination of their detectable characteristics. Peaks were quantified using area-

under-the-curve. Raw area counts for each metabolite in each sample were normalized to correct for variation resulting from instrument inter-day tuning differences by the median value for each run-day, therefore, setting the medians to 1.0 for each run. This preserved variation between samples but allowed metabolites of widely different raw peak areas to be compared on a similar graphical scale. Missing values were imputed with the observed minimum after normalization.

Table S1. Metabolite identity, subpathway, and superpathway of consensus weighted gene co-expression networks.

Metabolites	Super-pathway	Sub-pathway	Network Membership Score Male	Network Membership Score Female
**ME4 – Cellular stress response**				
taurine	Amino Acid	Methionine, Cysteine, SAM and Taurine Metabolism	0.94	0.92
glycerophosphoethanolamine	Lipid	Phospholipid Metabolism	0.94	0.92
phosphoethanolamine	Lipid	Phospholipid Metabolism	0.94	0.92
1-(1-enyl-palmitoyl)-GPE (P-16:0)*	Lipid	Lysoplasmalogen	0.91	0.89
1-stearoyl-2-oleoyl-GPS (18:0/18:1)	Lipid	Phosphatidylserine (PS)	0.90	0.90
1-(1-enyl-stearoyl)-GPE (P-18:0)*	Lipid	Lysoplasmalogen	0.89	0.88
choline phosphate	Lipid	Phospholipid Metabolism	0.88	0.87
inosine 5'-monophosphate (IMP)	Nucleotide	Purine Metabolism, (Hypo)Xanthine/Inosine containing	0.87	0.84
sphinganine	Lipid	Sphingolipid Synthesis	0.85	0.81
beta-citrylglutamate	Amino Acid	Glutamate Metabolism	0.85	0.87
**ME5 - Energetics**				
dihomo-linolenoylcarnitine (C20:3n3 or 6)*	Lipid	Fatty Acid Metabolism(Acyl Carnitine)	0.83	0.75
5-oxoproline	Amino Acid	Glutathione Metabolism	0.81	0.83
linoleoylcarnitine (C18:2)*	Lipid	Fatty Acid Metabolism(Acyl Carnitine)	0.81	0.77
oleoylcarnitine (C18:1)	Lipid	Fatty Acid Metabolism(Acyl Carnitine)	0.80	0.78
arachidonoylcarnitine (C20:4)	Lipid	Fatty Acid Metabolism(Acyl Carnitine)	0.77	0.75
cys-gly, oxidized	Amino Acid	Glutathione Metabolism	0.74	0.71
adrenoylcarnitine (C22:4)*	Lipid	Fatty Acid Metabolism(Acyl Carnitine)	0.71	0.67
ornithine	Amino Acid	Urea cycle; Arginine and Proline Metabolism	0.69	0.73
dihomo-linoleoylcarnitine (C20:2)*	Lipid	Fatty Acid Metabolism(Acyl Carnitine)	0.68	0.63
palmitoylcarnitine (C16)	Lipid	Fatty Acid Metabolism(Acyl Carnitine)	0.68	0.65
**ME7 - Androgen hormones**				
androstenediol (3beta,17beta) disulfate (2)	Lipid	Androgenic Steroids	0.88	0.90
androstenediol (3beta,17beta) disulfate (1)	Lipid	Androgenic Steroids	0.82	0.83
androstenediol (3beta,17beta) monosulfate (1)	Lipid	Androgenic Steroids	0.81	0.82
dehydroisoandrosterone sulfate (DHEA-S)	Lipid	Androgenic Steroids	0.81	0.87
21-hydroxypregnenolone disulfate	Lipid	Pregnenolone Steroids	0.81	0.84
Unannotated	--	--	0.79	0.78
Unannotated	--	--	0.77	0.78
Unannotated	--	--	0.77	0.76
androstenediol (3alpha, 17alpha) monosulfate (3)	Lipid	Androgenic Steroids	0.77	0.73
Unannotated	--	--	0.77	0.79

Table S1. Continued.

Metabolites	Super-pathway	Sub-pathway	Network Membership Score Male	Network Membership Score Female
**ME8 - Lysophospholipids**				
1-oleoyl-GPC (18:1)	Lipid	Lysophospholipid	0.86	0.87
1-stearoyl-GPC (18:0)	Lipid	Lysophospholipid	0.83	0.85
1-linoleoyl-GPC (18:2)	Lipid	Lysophospholipid	0.84	0.84
1-palmitoyl-GPC (16:0)	Lipid	Lysophospholipid	0.81	0.83
1-oleoyl-GPE (18:1)	Lipid	Lysophospholipid	0.84	0.82
1-stearoyl-GPE (18:0)	Lipid	Lysophospholipid	0.81	0.79
1-(1-enyl-palmitoyl)-GPC (P-16:0)*	Lipid	Lysoplasmalogen	0.76	0.77
1-linoleoyl-GPE (18:2)*	Lipid	Lysophospholipid	0.79	0.76
1-linolenoyl-GPC (18:3)*	Lipid	Lysophospholipid	0.76	0.74
2-palmitoyl-GPC (16:0)*	Lipid	Lysophospholipid	0.72	0.74
**ME9 - Microbial & Aromatic Amino Acid Metabolism**				
phenylacetylglutamine	Peptide	Acetylated Peptides	0.90	0.89
p-cresol glucuronide*	Amino Acid	Tyrosine Metabolism	0.79	0.79
Unannotated	--	--	0.77	0.79
phenylacetylcarnitine	Peptide	Acetylated Peptides	0.75	0.71
Unannotated	--	--	0.75	0.76
Unannotated	--	--	0.74	0.75
phenylacetate	Amino Acid	Phenylalanine Metabolism	0.71	0.71
Unannotated	--	--	0.69	0.71
Unannotated	--	--	0.69	0.74
Unannotated	--	--	0.62	0.75
3-indoxyl sulfate	Amino Acid	Tryptophan Metabolism	0.61	0.67
**ME11 - Diacylglycerols**				
palmitoyl-linoleoyl-glycerol (16:0/18:2) [2]*	Lipid	Diacylglycerol	0.84	0.78
palmitoyl-linoleoyl-glycerol (16:0/18:2) [1]*	Lipid	Diacylglycerol	0.80	0.69
oleoyl-linoleoyl-glycerol (18:1/18:2) [2]	Lipid	Diacylglycerol	0.79	0.74
oleoyl-linoleoyl-glycerol (18:1/18:2) [1]	Lipid	Diacylglycerol	0.79	0.72
diacylglycerol (16:1/18:2 [2], 16:0/18:3 [1])*	Lipid	Diacylglycerol	0.78	0.80
palmitoleoyl-arachidonoyl-glycerol (16:1/20:4) [2]*	Lipid	Diacylglycerol	0.77	0.75
linoleoyl-linolenoyl-glycerol (18:2/18:3) [2]*	Lipid	Diacylglycerol	0.76	0.77
linoleoyl-arachidonoyl-glycerol (18:2/20:4) [2]*	Lipid	Diacylglycerol	0.75	0.71
oleoyl-arachidonoyl-glycerol (18:1/20:4) [2]*	Lipid	Diacylglycerol	0.74	0.71
oleoyl-linolenoyl-glycerol (18:1/18:3) [2]*	Lipid	Diacylglycerol	0.73	0.73

Table S1. Continued.

Metabolites	Super-pathway	Sub-pathway	Network Membership Score	
			Male	Female
**ME13 - Bile Acid Metabolism**				
glycochenodeoxycholate	Lipid	Primary Bile Acid Metabolism	0.87	0.84
glycocholate	Lipid	Primary Bile Acid Metabolism	0.86	0.85
taurochenodeoxycholate	Lipid	Primary Bile Acid Metabolism	0.85	0.83
glycohyocholate	Lipid	Secondary Bile Acid Metabolism	0.78	0.74
taurocholate	Lipid	Primary Bile Acid Metabolism	0.78	0.76
glyco-beta-muricholate**	Lipid	Primary Bile Acid Metabolism	0.75	0.57
glycoursodeoxycholate	Lipid	Secondary Bile Acid Metabolism	0.74	0.71
glyco-alpha-muricholate**	Lipid	Primary Bile Acid Metabolism	0.71	0.63
taurodeoxycholate	Lipid	Secondary Bile Acid Metabolism	0.69	0.69
tauro-beta-muricholate	Lipid	Primary Bile Acid Metabolism	0.67	0.57

*Indicates tier 2 identification in which no commercially available authentic standards could be found, however annotated based on accurate mass, spectral and chromatographic similarity to tier 1 identified compounds.
** Indicates tier 2 identification in which no standard is available, but is annotated based on accurate mass, spectral and chromatographic similarity to tier 1 identified compounds.
